# The mitochondrial genome of *Argyra pingwuensis* (Diptera: Dolichopodidae)

**DOI:** 10.1080/23802359.2019.1627937

**Published:** 2019-07-16

**Authors:** Qilemoge, Chunmin Zhang, Dongyue He, Junhua Zhang, Ding Yang

**Affiliations:** a College of Plant Protection, China Agricultural University, Beijing, China;; b Institute of Plant Quarantine, Chinese Academy of Inspection and Quarantine, Beijing, China

**Keywords:** Mitochondrial genome, Diaphorinae, phylogenetics

## Abstract

The long-legged fly *Argyra pingwuenesis* Qilemoge, Wang et Yang (Genbank accession number: MK905737) belongs to the subfamily Diaphorinae of Dolichopodidae. The mitogenome of *A. pingwuenesis* was sequenced, the first representative of the mitogenome of the subfamily. The mitogenome is 15,859 bp long, consisting of 13 protein-coding genes, 2 rRNAs, and 22 transfer RNAs. All genes have the similar locations and strands with that of other published species of Dolichopodidae. The nucleotide composition biases toward A and T, which together made up 74.6% of the entirety. Bayesian inference analysis strongly supported the monophyly of Dolichopodidae. It suggested that subfamily Diaphorinae is the sister group of subfamily Rhaphiinae.

## Introduction

Diaphorinae belongs to the family Dolichopodidae, and the *Argyra* Macquart, 1834 is a larger genus of Diaphorinae with 120 extant species and 3 fossil species known from the world (Yang et al. 2006; 2011; Grichanov 2017; Qilemoge, Wang, et al. 2018).

The adult specimens of *Argyra pingwuensis* (Genbank accession number: MK905737) used for this study were collected from Pingwu (32°91′52″ N, 104°16′38″ E, 2930 m) of Sichuan Province in China in 2016. The specimens of *A. pingwuensis* were deposited in the Entomological Museum of China Agricultural University (CAU). The total genomic DNA was extracted from the whole body (except the head) of the specimen using the QIAamp DNA Blood Mini Kit (Qiagen, Germany) and stored at −20 °C until needed. The nearly complete mitogenome of *A. pingwuensis* is 15,859 bp. It encoded 13 PCGs, 22 tRNA genes, 2 rRNA genes and the control region could not be sequenced entirely in this study, and were similar with related reports before (Kang et al. 2016; Li et al. 2016; Li et al. 2017; Qilemoge, Gao, et al. 2018; Qilemoge, Zhang, et al. 2018; Wang, Ding, et al. 2016; Wang, Li, et al. 2016; Wang, Liu, et al. 2016; Wang, Wang, et al. 2016; Zhou et al. 2017; Hou et al. 2019). The nucleotide composition of the mitogenome was biased toward A and T, with 74.6% of A + T content (A = 38.4%, T = 36.2%, C = 15.5%, G = 10.0%). The A + T content of PCGs, tRNAs, and rRNAs is 71.9, 75.7, and 78.8%, respectively. The total length of all 13 PCGs of *A. pingwuensis* is 11,186 bp. All PCGs in *A. pingwuensis* utilize the conventional translational start codons for invertebrate mtDNA. For example, six PCGs (*COII*, *COIII*, *ATP6*, *ND4, ND4L*, and *CYTB*) initiated with ATG codons, four PCGs (*ND2*, *ND3, ND5*, and *ATP8*) initiated ATT codons, two PCGs (*ND1* and *ND6*) initiated ATA codons, *COI* initiated with TCG as a start codon. Eight PCGs used the typical termination codons TAA and three PCGs (*ND2*, *ND3*, and *CYTB*) used TAG in *A. pingwuensis*.

**Figure 1.**
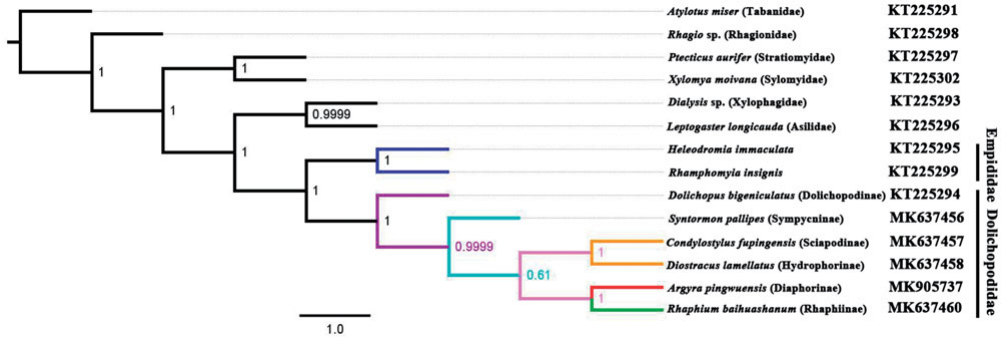
The phylogenetic tree of Bayesian interface analysis based on 13 PCGs and 2 rRNAs from 14 species.

Phylogenetic analysis was performed based on the nucleotide sequences of 13 PCGs and 2 rRNAs from 14 Diptera species. Bayesian (BI) analysis (Figure 1) showed that monophyletic Empidoidea was assigned to be the sister group to the clade of Xylophagidae and Asilidae. For the phylogeny of Empidoidea, monophyletic Empididae was assigned to the sister to monophyletic Dolichopodidae. For the phylogeny of Dolichopodidae, Diaphorinae was assigned to the sister of Rhaphiinae. The phylogenetic relationship within Empidoidea is very clear: Empididae + (Dolichopodinae + (Sympycninae + ((Sciapodinae + Hydrophorinae) + (Diaphorinae + Rhaphiinae)))). The position of Diaphorinae was also supported by the morphological study (Yang et al. 2011). The mitogenome of *A. pingwuensis* could provide important information for the further studies of Dolichopodidae phylogeny.
